# The Extract from the Stem and Leaf of *Paeonia lactiflora* Pall Has Demonstrated an Anti-Oxidative Stress Effect in Alleviating Diarrhea by Regulating the Gut-Liver Axis

**DOI:** 10.3390/antiox14050592

**Published:** 2025-05-15

**Authors:** Ming-Hua Wang, Ling Liu, Jun Li, Wei-Wei Zhou, Wei Tian, Jin-Hua Zhao, Xiu-Mei Li

**Affiliations:** Key Laboratory of Feed Biotechnology, Ministry of Agriculture and Rural Affairs, Institute of Feed Research of CAAS, Beijing 100081, China; 18395733955@163.com (M.-H.W.); 18630616370@163.com (L.L.); lijun@caas.cn (J.L.); zhouweiwei@caas.cn (W.-W.Z.); ttww1234@163.com (W.T.); zhaojinhua@caas.cn (J.-H.Z.)

**Keywords:** *Paeonia lactiflora* pall, stem and leaf, extract, broiler, oxidative stress, diarrhea

## Abstract

This study investigated the preventive effects and mechanisms of *Paeonia lactiflora* pall stem and leaf extract (PLE) on oxidative stress-induced diarrhea in broilers, using a Diquat (DQ)-induced model. Results indicated that PLE significantly improved growth performance, increased average daily gain (ADG), reduced feed-to-gain ratio (F/G), and enhanced liver and kidney indices. PLE alleviated DQ-induced oxidative stress diarrhea by reducing the diarrhea rate by 63.84%, upregulating mRNA expression of *MUC2*, *Claudin-1*, *ZO-1*, and *Occludin*, and decreasing AST and ALT activities in serum. Additionally, PLE increased levels of CAT, SOD, GSH-Px, and GSH while reducing PCO and MDA levels in serum, intestine, and liver tissues. Furthermore, PLE increased acetic acid content and decreased propionic acid, butyric acid, and isobutyric acid contents. PLE also altered gut microbiota by up-regulated Bacteroidetes and *Barnesiella* and down-regulated Firmicutes and *unclassified_o__Eubacteriales*. Network pharmacology suggested that PLE acts via the PI3K-Akt-Nrf2 pathway, confirmed by up-regulated mRNA expression of PI3K, AKT, Nrf2, NQO1, and HO-1, and down-regulated Keap1 in intestinal and liver tissues. Correlation analysis revealed significant associations between *Barnesiella* and *unclassified_o__Eubacteriales* with short-chain fatty acids and PI3K-Akt-Nrf2 pathway-related genes. Thus, PLE prevents and alleviates oxidative stress-induced diarrhea in broilers by modulating the PI3K-Akt-Nrf2 pathway, regulating gut microbiota, and influencing short-chain fatty acids.

## 1. Introduction

Diarrhea is a prevalent and recurrent disease in livestock and poultry, caused by a variety of factors, with high morbidity and mortality rates that lead to significant economic losses [1]. In recent years, oxidative stress (OS) has been identified as a critical factor in the pathogenesis of diarrhea in livestock and poultry. Under OS conditions, intestinal epithelial cells experience oxidative damage, which inhibits the expression of tight junction proteins, compromises the integrity of the intestinal barrier, and significantly increases intestinal permeability, thereby facilitating the invasion of harmful substances [2]. Moreover, the excessive accumulation of free radicals disrupts the intestinal redox balance, suppresses the growth and activity of beneficial bacteria, and promotes the proliferation of harmful bacteria. This dysbiosis directly affects the composition of intestinal metabolites, particularly reducing the production of short-chain fatty acids (SCFAs), which further exacerbates intestinal damage. When impaired intestinal barrier function coexists with dysbiosis, harmful substances can easily penetrate into systemic circulation, not only causing direct intestinal tissue damage but also accelerating intestinal peristalsis, ultimately inducing diarrhea symptoms [3]. The study by Jeon et al. [4] also showed that OS triggered cellular damage and led to intestinal inflammation and diarrhea symptoms. Therefore, effective control and mitigation of OS can significantly reduce the incidence of diarrhea in livestock and poultry, thus improving the economic outcomes of livestock farming and the health status of animals.

*Paeonia lactiflora* pall (*Paeonia lactiflora*) is a perennial herb from the family Paeoniaceae, with its medicinal part being the dried root, commonly referred to as “red peony root” in traditional Chinese medicine. However, in practice, the stems and leaves of *Paeonia lactiflora* are often considered waste, leading to the underutilization of these potential resources. Studies have revealed that the stems and leaves of *Paeonia lactiflora* are rich in antioxidant components, including glycosides (e.g., paeoniflorin, paeoniflorin lactone glycosides), polyphenols (e.g., gallic acid, methyl gallate, ethyl gallate), and flavonoids (e.g., kaempferol, chrysin) [5,6]. Although studies on the stems and leaves of *Paeonia lactiflora* to alleviate Diquat (DQ)-induced oxidative stress diarrhea have not been reported, their active constituents have been shown to mitigate OS through multiple signal pathway. For instance, Yuan et al. [7] reported that kaempferol activates the *Nrf2-SLC7A11-GPX4* signaling pathway, enhances antioxidant capacity, and inhibits lipid peroxidation in OGD/R-treated neurons. Similarly, Jia et al. [8] demonstrated that paeoniflorin reduces malondialdehyde (MDA) levels while increasing the activities of superoxide dismutase (SOD), catalase (CAT), and glutathione peroxidase (GSH-Px). Additionally, Li et al. [9] found that paeoniflorin protects cardiomyocytes from oxidative damage by reducing the production of reactive oxygen species (ROS).

Therefore, in this study, we investigated the preventive diarrhea effect of PLE based on the DQ-induced OS model in broilers. First, we detected and analyzed the effects of PLE on growth performance, oxidative stress biomarkers, short chain fatty acids, and intestinal flora. Subsequently, we predicted the signaling pathway and validated it through which PLE alleviates oxidative stress-induced diarrhea. Finally, we analyzed the correlation between each detection index. Thus, the mechanism by which PLE prevents oxidative stress diarrhea in broilers was elucidated. This study provides a theoretical basis for PLE application in treating livestock and poultry oxidative stress diarrhea.

## 2. Materials and Methods

### 2.1. Plant Material

The dried stems and leaves of Paeonia lactiflora pall from Hulunbeier. All specimens were authenticated by Xiumei Li, Feed Research Institute, Chinese Academy of Agricultural Sciences, Beijing, China.

### 2.2. Extract Preparation

The dried stems and leaves of Paeonia lactiflora pall were pulverized and sieved through a 60-mesh sieve, and then subjected to ultrasonic extraction under the following conditions: a 50% (*v*/*v*) ethanol/water solution, a 1:30 (g/mL) ratio of material to liquid, a temperature of 55 °C, and 500 W ultrasonic power for 25 min. The extract was centrifuged at 7500 rpm for 15 min, and the supernatant liquid was concentrated and freeze-dried to obtain PLE for subsequent use.

### 2.3. Animal Testing and Grouping

A total of 144 one-day-old male Arbor Acres (AA) broilers (weighing 35.3 ± 2.1 g) were obtained from Shunyi Hatchery in Beijing, China. The broilers were housed in well-ventilated, sanitized poultry pens with ad libitum access to water and feed. After a 7-day acclimation period, randomization was performed at 09:00 on day 8 using a computer-based random number generator (Microsoft Excel’s RAND function). The birds were first sorted by identification number, and each was assigned a random value. Based on these values, the broilers were stratified into three groups: the lowest third assigned to the control group (CON), the middle third to the DQ group, and the highest third to the PLE group. Each treatment group comprised 3 replicates (16 birds per replicate), with an average body weight of 209.4 ± 2.3 g at allocation. The CON and DQ groups received the basal diet, whereas the PLE group was fed the basal diet supplemented with 0.3% PLE. The experimental period lasted 28 days. On day 28 at 07:00, the CON group was injected with 0.9% saline, and the broilers in the DQ and PLE groups were injected intraperitoneally with 10 mg/kg body weight (bw) of DQ. The broiler chickens were housed in single-layer cages with a 23-h light:1-h dark photoperiod. Throughout the experimental period, they had free access to feed and clean drinking water. After one week of rearing, the room temperature was maintained at approximately 35 °C and then gradually reduced to around 20 °C until the end of the experiment. All broilers were treated in compliance with the Ethical Committee for Animal Research of the Institute of Feed Research of CAAS (Assurance NO. IFR-CAAS-20240321).

### 2.4. Sample Collection

On day 29 at 07:00, six broilers per group were selected randomly using the same computer-generated randomization protocol (Microsoft Excel RAND function) described in Section 2.3. First, broilers in each group were renumbered, and new random values were assigned. The six broilers with the lowest random values were selected for venous blood collection and serum separation. The selected broilers were then euthanized. Immediately after euthanasia, liver, kidney, spleen, thymus, and bursa of Fabricius tissues were harvested. Jejunum tissue was simultaneously excised and rinsed with 0.9% saline. Portions of jejunal and liver tissues were fixed in 4% paraformaldehyde for paraffin embedding, while the remaining samples were flash-frozen in aluminum foil and stored at −80 °C for subsequent analysis. Cecal contents were aseptically transferred to sterile cryovials and snap-frozen in liquid nitrogen for gut microbiota and SCFA analysis.

### 2.5. Determination of Growth Performance and Organ Indices

Body weight and feed intake of the broilers were recorded on days 1, 7, 14, 21, and 28 of the experiment to calculate the average daily gain (ADG) and average daily feed intake (ADFI). The ADFI and ADG were then used to calculate the feed-to-gain ratio (F/G) for each experimental stage.

After euthanizing the broilers, their organs, including the liver, spleen, kidney, bursa, and thymus, were carefully excised and weighed. The organ index was calculated using the formula: organ index = organ weight/live weight.

### 2.6. Calculation of Diarrhea Rate

The diarrhea rate in the broilers was calculated by assessing the degree of anal filth and the percentage of diarrheal feces in the fecal tray, using the following formula: dirty anus rate (%) = (number of broilers with dirty anuses/total number of broilers) × 100%

Percentage of fecal tray diarrhea (%) = (number of diarrhea-like feces/total feces in the fecal tray) × 100%

Broiler diarrhea rate (%) = (percentage of anal filth + percentage of fecal tray diarrhea)/2 × 100%

### 2.7. Morphologic Observation of Jejunal and Liver Tissues

After preparing HE-stained sections of paraffin-embedded liver and duodenal tissues, histomorphological observations were made using a light microscope. Morphological parameters, including villus height (VH), crypt depth (CD), and the villus height/crypt depth ratio (VH/CD), were calculated from the acquired images.

### 2.8. Biochemical Analysis

The activities of liver function-related enzymes, including aspartate transaminase (AST, C010-2-1) and alanine aminotransferase (ALT, C009-2-1), in serum were determined using colorimetric kits from Nanjing Jiancheng Bioengineering Institute. Total antioxidant capacity (T-AOC, S0116), superoxide dismutase (SOD, S0101S), catalase (CAT, S0051), and glutathione peroxidase (GSH-Px, S0058) in broiler serum were detected using kits from Beyotime Biotechnology. Glutathione (GSH, A006-1-1), protein carbonyl content (PCO, A087-1-1), and malondialdehyde (MDA, A003-1-2) in broiler serum, intestine, and liver tissue were detected using the colorimetric kits from Nanjing Jianjian Bioengineering Institute.

### 2.9. Determination of Related mRNA Expression Levels

The real-time fluorescence quantitative PCR (RT-qPCR) method was used for analysis [10]. Total RNA was extracted from broiler jejunum and liver tissues using TRIzol reagent, and reverse transcription was performed following the instructions of the TransScript R First-Strand cDNA Synthesis SuperMix. The sequences of the differential gene primers are listed in Table 1. The RT-qPCR program consisted of an initial denaturation at 95 °C for 10 min, followed by 40 cycles of 95 °C for 15 s and 60 °C for 1 min, with a final step of 95 °C for 15 s.

### 2.10. Detection of SCFAs in Cecum Contents

Weigh accurately 100.0 ± 50.0 mg of fecal sample into a 2 mL centrifuge tube, add 1.0 mL PBS solution, vortex-mix vigorously, and centrifuge at 10,000 rpm for 5 min. Transfer 1.0 mL supernatant to a 20 mL headspace vial, add 0.25 mL 15% methanolic sulfuric acid solution, and seal immediately. Standard solutions of acetic acid, propionic acid, isobutyric acid, butyric acid, isovaleric acid, and valeric acid were prepared at various concentrations for calibration. SCFAs were analyzed using an RB-614 column with the following temperature program: 60 °C for 3 min, ramped to 80 °C at 40 °C/min, then held at 80 °C for 2 min. The injector temperature was maintained at 200 °C with a split ratio of 20:1. Ultra-high-purity nitrogen served as the carrier gas with the following flow program: 2 mL/min for 3 min, 20 mL/min, then 3 mL/min for 3 min. The FID detector was set at 220 °C with tail gas flow of 30 mL/min, hydrogen flow rate of 30 mL/min and air flow rate of 300 mL/min. Headspace operating parameters were: oven temperature 80 °C, needle temperature 90 °C, valve temperature 100 °C, transfer line temperature 110 °C, equilibration time of 30 min, and 1.0 mL injection volume.

### 2.11. 16S rRNA Sequencing of Cecum Contents

In this study, we adopted the 16S rRNA sequencing method described by Ruan et al. [11]. The detailed steps are as follows: the total DNA was extracted from the collected cecal contents and processed with the E.Z.N.A.^®^ Soil DNA Kit (Omega Bio-tek, Norcross, GA, USA). The quality and concentration of the extracted DNA were verified by 1.0% agarose gel electrophoresis and a NanoDrop^®^ ND-2000 spectrophotometer(Thermo Fisher Scientific, Waltham, MA, USA). Amplification of the V3-V4 region of the 16S rRNA gene was performed using specific primers 338F and 806R. The polymerase chain reaction (PCR) was conducted under the following stringent conditions: pre-denaturation at 95 °C for 3 min, followed by 27 cycles of denaturation at 95 °C for 30 s, annealing at 55 °C for 30 s, and extension at 72 °C for 45 s, with a final extension at 72 °C for 10 min. PCR products were purified using 2% agarose gel electrophoresis and the AxyPrep DNA Gel Extraction Kit(AXYGEN Scientific, Union City, CA, USA), then quantified with the Quantus™ -ST Fluorometer(Promega Corporation, Madison, WI, USA). After equimolar mixing, the samples were sequenced in paired-end mode on Illumina MiSeq PE300 or NovaSeq PE250 platforms. Bioinformatics analysis of gut microbiota was conducted on the Major BioCloud platform, available at https://cloud.majorbio.com, (accessed on 12 May 2024).

### 2.12. Correlation Analysis

Bioinformatics analysis of gut microbiota was conducted on the Major BioCloud platform (https://cloud.majorbio.com, accessed on 12 May 2024). Microbial abundance (Top 10) was analyzed at the “Phylum/Genus” classification level, clustering by species. The Pearson algorithm was applied with a correlation coefficient threshold of 0.1 and a significance level of *p* < 0.05 to perform a correlation analysis between the abundance of intestinal flora and oxidative stress biomarkers. Furthermore, a correlation analysis of broilers’ gut microbiota was conducted using the Pearson algorithm. Data with a correlation coefficient > 0.1 and *p* < 0.05 were selected to construct a correlation network diagram.

### 2.13. Statistical Analysis

Data were summarized in Excel and expressed as mean ± SEM. ANOVA was conducted using GraphPad Prism 9 and SPSS 22.0, and graphs were generated to present the results. For alpha diversity analysis, non-normally distributed data were analyzed using the Kruskal–Wallis test. Beta diversity analysis was performed using the binary_jaccard algorithm to assess inter-sample variability by calculating distances and deriving beta values. Correlation analyses were conducted using Pearson’s correlation coefficient. Statistical significance was defined as *p* < 0.05, with *p* < 0.01 indicating high statistical significance.

## 3. Results

### 3.1. Effects of PLE on Broiler Growth Performance and Relative Organ Weights

As indicated in Table 2, there were no significant variations in ADG, ADFI, and F/G between the DQ and CON groups. However, in comparison to the DQ group, the PLE group exhibited a significant 7.61% increase in ADG (*p* < 0.01) and an 11.85% decrease in F/G (*p* < 0.01), with ADFI remaining constant. When compared to the CON group, the thymus index of the DQ group was reduced by 44.98% (*p* < 0.01), while no significant differences were noted in the liver index, kidney index, and bursa index. In contrast, the liver index, kidney index, and thymus index of the PLE group were elevated by 18.42%, 77.12%, and 59.93%, respectively (*p* < 0.001). Furthermore, PLE did not significantly impact the spleen index and bursa index of broilers.

### 3.2. Preventive Effect of PLE on Oxidative Stress Diarrhea in Broilers

#### 3.2.1. Effect of PLE on Diarrhea Rate in Broilers

**Figure 1:**
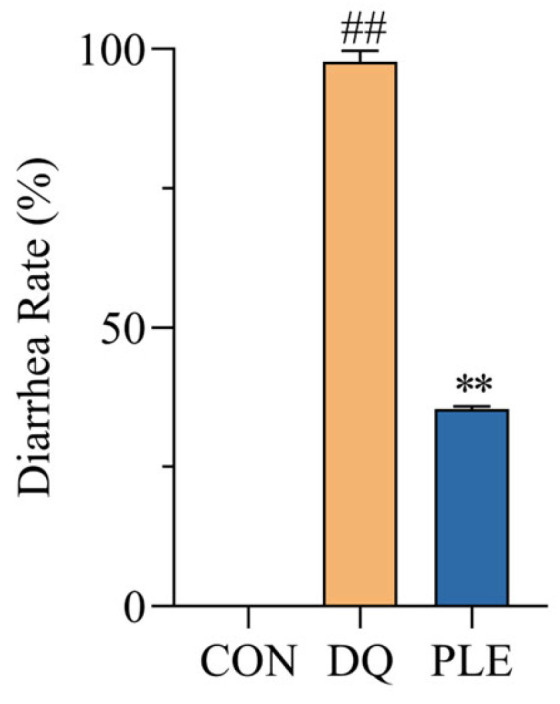
Effect of PLE on diarrhea rate of broilers. Compared with the CON group, ^##^ *p* < 0.01; compared with the DQ group, ** *p* < 0.01.

As illustrated in Figure 1, the diarrhea rate in the DQ group reached 97.7%, significantly higher than that of the CON group. In contrast, the PLE group experienced a 63.84% reduction in diarrhea rates compared to the DQ group (*p* < 0.01). These results demonstrate that PLE effectively prevents DQ-induced oxidative stress diarrhea in broilers.

#### 3.2.2. Effect of PLE on DQ-Induced Oxidative Stress Damage in Serum

**Figure 2:**
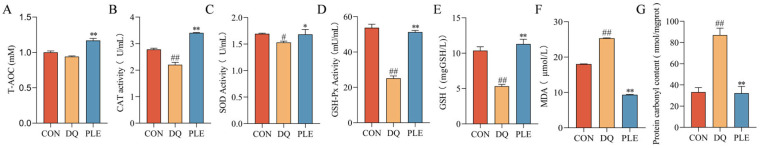
Preventive effect of PLE on oxidative stress damage in serum. (**A**–**G**) T-AOC, CAT, SOD and GSH-Px activities, and GSH, MDA, and PCO contents in serum. Compared with the CON group, ^#^ *p* < 0.05, ^##^ *p* < 0.01; compared with the DQ group, * *p* < 0.05, ** *p* < 0.01.

Depicted in Figure 2, compared with the DQ group, the PLE group significantly decreased MDA and PCO contents (*p* < 0.01), while increasing T-AOC and GSH contents as well as CAT, SOD, and GSH-Px activities in serum (*p* < 0.05 or *p* < 0.01). These results indicate that DQ-induced oxidative stress aggravates serum oxidative damage, while PLE supplementation effectively alleviates this damage by restoring oxidative stress biomarkers, improving the antioxidant defense system, and consequently reducing oxidative stress-associated diarrhea.

#### 3.2.3. Effect of PLE on DQ-Induced Oxidative Stress Damage in Liver Tissues

**Figure 3:**
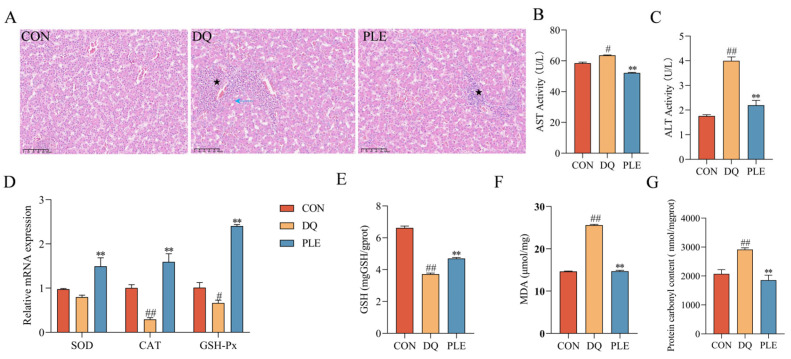
Effect of PLE on DQ-induced oxidative stress damage in the liver tissue. (**A**) Liver histomorphology was observed by HE staining; black pentagrams indicate inflammatory cell infiltration, and blue arrows indicate hepatocyte degeneration and necrosis. (**B**,**C**) AST and ALT activities in serum. (**D**) mRNA expression levels of *SOD*, *CAT*, and *GSH-Px* in liver tissue. (**E**–**G**) Levels of GSH, MDA, and PCO in liver tissue. Differences between groups were analyzed by one-way ANOVA. Compared with the CON group, ^#^ *p* < 0.05, ^##^ *p* < 0.01; compared with the DQ group, ** *p* < 0.01.

To evaluate the preventive effect of PLE on liver oxidative damage, histopathological examination and biochemical analysis of key oxidative stress markers were conducted. Depicted in Figure 3, HE staining revealed disorganization of liver tissues in the DQ group compared to the CON group, including irregular hepatic cords, hepatocyte degeneration and necrosis (blue ↑), marked dilation of hepatic sinusoids, and multiple inflammatory cell foci in the sinusoids (★). Compared to the DQ group, the liver tissues in the PLE group showed no degeneration or necrosis of hepatocytes, mild dilation of hepatic sinusoids, and only a small number of inflammatory cell foci in the sinusoids (★), indicating improved liver conditions. Biochemical analysis showed that serum AST and ALT activities were significantly elevated in the DQ group compared to the CON group (*p* < 0.05 or *p* < 0.01). However, PLE effectively reduced these activities (*p* < 0.05 or *p* < 0.01), suggesting a protective effect of PLE on liver function. In addition, the liver tissues of the DQ group showed significantly increased MDA and PCO contents (*p* < 0.05) and significantly decreased GSH content, down-regulated the mRNA expression levels of *CAT* and *GSH-Px* (*p* < 0.05 or *p* < 0.01), indicating a state of severe oxidative stress. In contrast, PLE significantly reduced MDA and PCO contents while increasing GSH content and up-regulated the mRNA expression levels of *SOD*, *CAT*, and *GSH-Px* (*p* < 0.01), suggesting that PLE effectively inhibited free radical accumulation, improved endogenous antioxidant capacity, and alleviated hepatic oxidative damage. In conclusion, PLE exhibited a significant protective effect against hepatic oxidative damage by improving histopathological conditions and enhancing the antioxidant defense system.

#### 3.2.4. Effect of PLE on DQ-Induced Oxidative Stress Damage in the Intestinal Tissue

**Figure 4:**
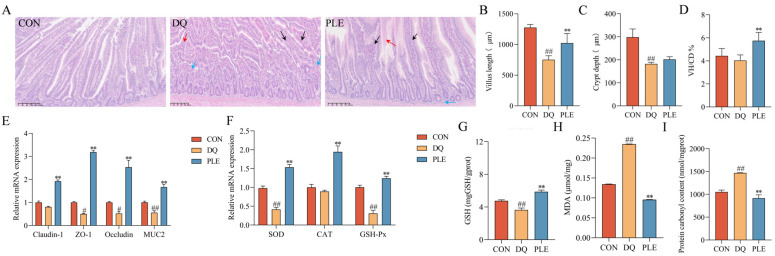
Effects of PLE on intestinal barrier function in broilers. (**A**) Intestinal histological images stained with hematoxylin and eosin (HE), where black arrows indicate shortened jejunal villi, red arrows indicate degeneration and necrosis of mucosal epithelial cells, and blue arrows indicate inflammatory cell infiltration. (**B**–**D**) Jejunal villus length, crypt depth, and the villus length/crypt depth ratio (VH/CD). (**E**) mRNA expression levels of *Claudin-1*, *ZO-1*, *Occludin*, and *MUC2* in the jejunum. (**F**) mRNA expression levels of *SOD*, *CAT*, and *GSH-Px* in intestinal tissues. (**G**–**I**) GSH, MDA, and PCO contents in liver tissues. Differences between groups were analyzed by one-way ANOVA. Compared with the CON group, ^#^ *p* < 0.05, ^##^ *p* < 0.01; compared with the DQ group, ** *p* < 0.01.

OS disrupts the body’s antioxidant balance, causing lipid peroxidation, oxidative protein damage, and DNA damage, which result in the excessive accumulation of ROS [12]. Such oxidative damage impairs intestinal barrier integrity and increases mucosal permeability, ultimately contributing to diarrhea pathogenesis [2]. Our results are shown in Figure 4. The results of HE staining revealed that in the DQ group, the jejunal villi were shortened and disorganized, with necrotic mucosal epithelial cells shed into the lumen and significant infiltration of inflammatory cells in the lamina propria. In contrast, the PLE group exhibited less severe histopathological changes, characterized by slight villus disorganization and limited inflammatory cell infiltration. Additionally, compared with the CON group, the DQ group showed a 38.99% decrease in crypt depth, a 41.02% decrease in villus length, and a 9.02% reduction in the villus length-to-crypt depth ratio (*p* < 0.01). In the PLE group, crypt depth and villus length increased significantly by 10.86% and 30.73%, respectively, compared with the DQ group. The villus length-to-crypt depth ratio also increased by 42.52%, approaching CON group levels (*p* < 0.01). These findings highlight the significant role of PLE in preserving the villus length-to-crypt depth ratio, suggesting its ability to maintain intestinal structural integrity. Moreover, the results of intestinal barrier-related gene expression showed that, compared with the CON group, the DQ group significantly down-regulated the mRNA expression levels of *MUC2*, *Claudin-1*, *ZO-1*, and *Occludin* (*p* < 0.05 or *p* < 0.01). In contrast, the PLE group significantly up-regulated the expression of these genes compared with the DQ group (*p* < 0.01). It is suggested that PLE improves intestinal barrier function and enhances gut health. In addition, the intestinal tissues of the DQ group showed significantly higher MDA and PCO contents (*p* < 0.01) and significantly lower *GSH* content and down-regulated the mRNA expression levels of *SOD* and *GSH-Px* (*p* < 0.01) compared to the PLE group, which exhibited significantly lower MDA and PCO contents (*p* < 0.01) and significantly up-regulated the mRNA expression levels of *SOD*, *CAT*, and *GSH-Px* as well as GSH content (*p* < 0.01).

#### 3.2.5. Effect of PLE on the Content of SCFAs

**Figure 5:**
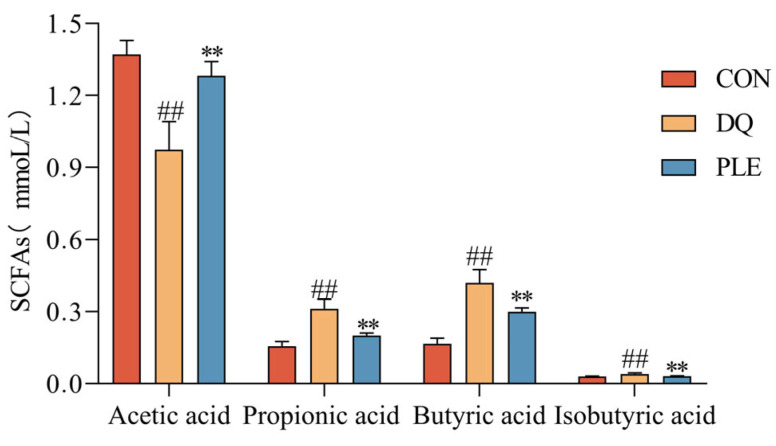
Effect of PLE on the content of SCFAs. Differences between groups were analyzed by one-way ANOVA. Compared with the CON group, ^##^ *p* < 0.01; compared with the DQ group, ** *p* < 0.01.

SCFAs are products of the fermentation of dietary fiber and other undigested carbohydrates by intestinal microorganisms, mainly including acetic acid, propionic acid, and butyric acid. These metabolites play a crucial role in maintaining intestinal health and overall metabolic homeostasis, mitigate oxidative damage in the body and alleviate OS-induced diarrhea by inhibiting ROS production [13]. To evaluate the changes in gut microbial metabolites, the contents of SCFAs were analyzed across different treatment groups. Depicted in Figure 5, compared to the CON group, the DQ group exhibited a significant reduction in acetic acid content and a marked increase in propionic acid, butyric acid, and isobutyric acid contents (*p* < 0.01). In contrast, the PLE significantly increased the content of acetic acid and decreased the contents of propionic acid, butyric acid, and isobutyric acid compared to the DQ group (*p* < 0.01). These findings suggest that PLE may exert anti-oxidative stress diarrhea effects through modulation of SCFAs.

#### 3.2.6. Effects of PLE on Gut Microbiota

**Figure 6:**
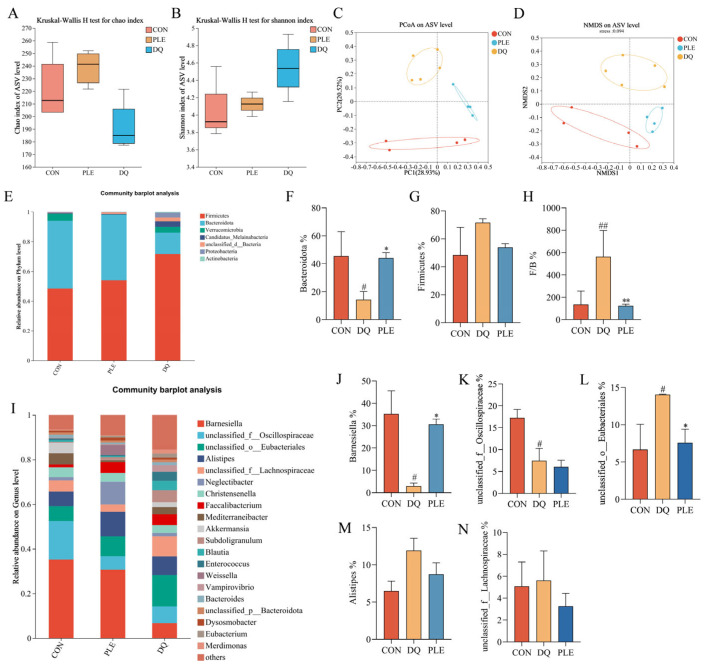
Effect of PLE on intestinal flora. (**A**) Chao index. (**B**) Shannon index. (**C**,**D**) PCoA and NMDS analysis based on ASV level. (**E**) Bacterial species composition at the phylum level in broilers. (**F**–**H**) Abundance of Bacteroidetes, Firmicutes, and the Firmicutes to Bacteroidetes ratio. (**I**) Bacterial species composition at the genus level in broilers. (**J**–**N**) Relative abundance of *Barnesiella*, *unclassified_f__Oscillospiraceae*, *unclassified_o__Eubacteriales*, Alistipes, and *unclassified_f__Lachnospiraceae*. Differences between groups were analyzed by one-way ANOVA. Compared with the CON group, ^#^ *p* < 0.05, ^##^ *p* < 0.01; compared with the DQ group, * *p* < 0.05, ** *p* < 0.01.

Floral imbalance, characterized by a reduction in beneficial bacteria and an increase in pathogenic bacteria, compromises the intestinal barrier and induces immune dysfunction, thereby elevating the risk of diarrhea [14]. Beneficial bacteria, such as lactobacilli and bifidobacteria, help prevent diarrhea by preserving intestinal barrier integrity and inhibiting pathogenic bacterial colonization [15]. Thus, maintaining intestinal flora balance is crucial for diarrhea prevention. Alpha diversity reflects the species diversity and richness within the samples. The Chao index, which measures species richness (Figure 6A), indicated no significant differences between the DQ and PLE groups compared to the CON group. Shannon’s index, which reflects species richness and evenness (Figure 6B), indicated that the PLE group had values closer to those of the CON group. However, no significant differences were observed among the DQ, PLE, and CON groups. Non-metric multidimensional scaling (NMDS, Figure 6C) and principal coordinate analysis (PCoA, Figure 6D) based on ASV levels revealed significant separation between treatment groups. Samples from the CON group were centrally located on the graph, indicating a stable microbial community structure. In contrast, the samples from the DQ group clustered together and showed significant deviation from the CON group, indicating that DQ treatment substantially altered the microbial community structure. Notably, the PLE group samples exhibited closer proximity to the CON group, demonstrating that the PLE group’s microbial community structure more closely resembled the control. These results indicate that PLE treatment effectively restores or maintains the microbial community structure.

Statistical analysis of species distribution and abundance at the phylum level revealed that the species with the highest relative abundance across the groups were mainly Firmicutes and Bacteroidetes, as depicted in Figure 6E. In the CON group, the relative abundance of Firmicutes and Bacteroidetes was 48.45% and 45.55%, respectively. However, in the DQ group, the relative abundance of Firmicutes up-regulated to 71.66%, while that of Bacteroidetes down-regulated to 14.34%. Dietary supplementation with PLE reversed this trend. As illustrated in Figure 6F,G, the relative abundance of Firmicutes was down-regulated, and the relative abundance of Bacteroidetes was significantly up-regulated in the PLE group compared to the DQ group. Furthermore, when the ratio of Firmicutes to Bacteroidetes (F/B) was calculated (Figure 6H), the F/B ratio was significantly up-regulated in the DQ group compared to the CON group, while the F/B ratio was significantly down-regulated in the PLE groups compared to the DQ group (*p* < 0.01).

Based on the species distribution at the phylum level, the relative abundance of the top 20 genera at the genus level was further analyzed, and the results are shown in Figure 6I. Among the groups, the genera that constituted a large proportion included *Barnesiella*, *unclassified_f__Oscillospiraceae*, *unclassified_o__Eubacteriales*, *Alistipes*, and *unclassified_f__Lachnospiraceae*. A significance analysis of the top five species between the groups was performed, as depicted in Figure 6J–N. Compared to the CON group, the DQ group exhibited a down-regulation in the relative abundance of *Barnesiella* and *unclassified_f__Oscillospiraceae*, while an up-regulation in the relative abundance of *unclassified_o__Eubacteriales* was observed (*p* < 0.05). However, in the PLE group, the relative abundance of *Barnesiella* was up-regulated, and the relative abundance of *unclassified_o__Eubacteriales* was down-regulated compared to the DQ group, with no significant difference observed in the relative abundance of *unclassified_f__Oscillospiraceae*. These results suggest that PLE reversed the DQ-induced down-regulation in the relative abundance of *Barnesiella* and up-regulation in the relative abundance of *unclassified_o__Eubacteriales*. Thus, PLE may exert its effects on oxidative stress-induced diarrhea by modulating the gut microbiota.

### 3.3. Mechanism of PLE in Preventing Oxidative Stress-Induced Diarrhea

#### 3.3.1. Network Pharmacology Predicts the Signaling Pathway of PLE Prevention of Oxidative Stress

Based on the prediction of antioxidant targets and pathways of PLE using network pharmacology, a total of 51 components in PLE were identified in a previous study (Table 3). The identified compounds comprised 13 polyphenols, 7 flavonoids, 7 organic acids, 11 glycosides, and 13 other compounds. Gene-level validation was subsequently conducted in accordance with the prediction results.

**Figure 7:**
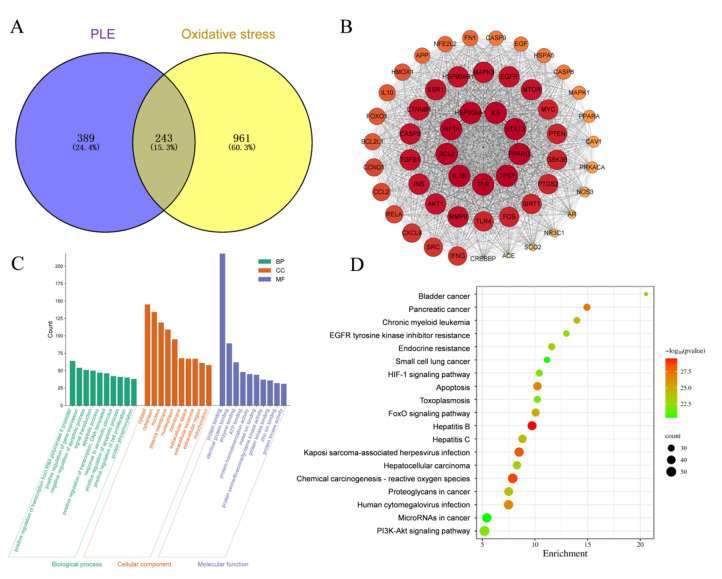
Network pharmacological predictions pathway. (**A**) Venn diagram of PLE component targets and oxidative stress intersection targets. (**B**) PPI network of PLE and oxidative stress. (**C**) GO biological functional enrichment analysis results. (**D**) KEGG pathway enrichment analysis results.

As depicted in Figure 7A, 632 targets of PLE component action and 1204 OS targets were identified. A Venn analysis of PLE action targets and OS targets revealed 243 overlapping targets. The intersection targets of PLE components and OS were utilized to construct a protein–protein interaction (PPI) network, as depicted in Figure 7B. This network comprises 54 nodes and 1313 edges. The network was analyzed using Cytoscape 3.8.0 software, and the top ten targets based on degree values were identified as *IL1β*, *HIF1A*, *IL6*, *STAT3*, *PPARG*, *TP53*, *TNF*, *BCL2*, *HSP90AA1*, and *AKT1*. The key antioxidant targets were imported into the DAVID database for Gene Ontology (GO) biological function and Kyoto Encyclopedia of Genes and Genomes (KEGG) pathway enrichment analysis. As illustrated in Figure 7C, a total of 432 GO terms were identified (*p* < 0.05). These included 120 entries for biological processes (BP), such as positive regulation of RNA polymerase II promoter transcription and positive regulation of gene expression; 127 entries for cellular components (CC), including cytoplasm, nucleus, and mitochondria; and 235 entries for molecular functions (MF), such as protein binding, enzyme binding, and ATP binding. KEGG enrichment analysis, using the DAVID database, identified 120 relevant signaling pathways (*p* < 0.05). Based on KEGG pathway enrichment analysis of the overlapping targets of PLE and OS, a total of 194 pathways were identified. The top 20 pathways with the most significant *p* values were visualized in a bubble plot, depicted in Figure 7D. The results indicated that the targets of PLE were primarily enriched in signaling pathways such as the HIF-1 signaling pathway, apoptosis, chemical carcinogenesis-reactive oxygen species (CCROS), FoxO signaling pathway, and *PI3K-Akt* signaling pathway. Among these, the PI3K-Akt signaling pathway plays a pivotal role in regulating cellular responses to OS. Activation of PI3K promotes cell survival and metabolism by phosphorylating and activating downstream Akt. This activation leads to the phosphorylation and activation of *Nrf2*, which dissociates from Keap1, thereby enhancing cellular antioxidant defense mechanisms. Based on these findings, it is hypothesized that PLE exerts its antioxidant effects by activating the *PI3K-Akt* signaling pathway, which subsequently influences the *Nrf2* signaling pathway, preventing oxidative stress-induced diarrhea.

#### 3.3.2. Validation of Key Targets of the *PI3K-Akt-Nrf2* Signaling Pathway

**Figure 8:**
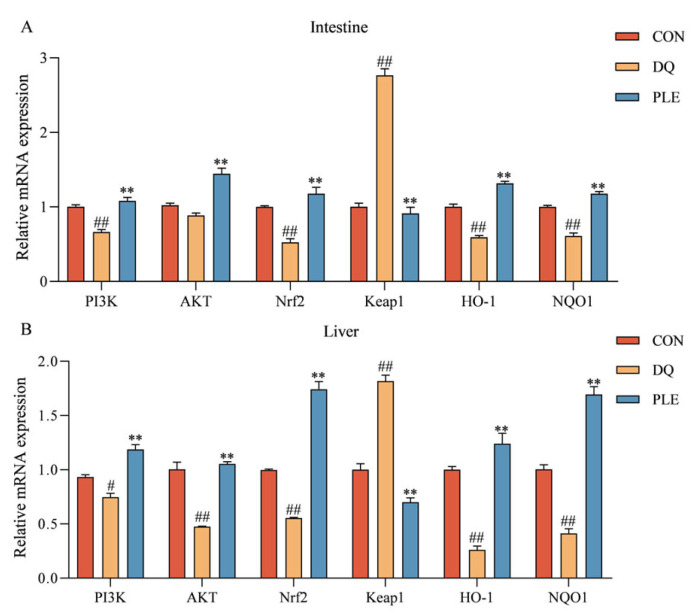
The impact of PLE on the mRNA expression levels of the *PI3K-AKT-Nrf2-Keap1* signaling pathway and downstream antioxidant-related genes in broiler intestinal tissues (**A**) and liver tissues (**B**). Group differences were analyzed using one-way ANOVA. Compared with the CON group, ^#^ *p* < 0.05, ^##^ *p* < 0.01; compared with the DQ group, ** *p* < 0.01.

Figure 8 depicts the impact of various treatments on the mRNA expression levels of genes within the PI3K-Akt-Nrf2-Keap1 signaling pathway and downstream antioxidant-related genes in broiler intestinal and liver tissues. Compared to the CON group, the DQ group showed a significant up-regulation of *Keap1* mRNA expression levels (*p* < 0.01), and a significant down-regulation of *PI3K*, *AKT*, *Nrf2*, *HO-1*, and NQO1 mRNA expression levels (*p* < 0.01 or *p* < 0.05). Conversely, the PLE group markedly reversed these trends, with a pronounced down-regulation in Keap1 mRNA expression levels and a significant increase in the mRNA expression levels of *PI3K*, *AKT*, *Nrf2*, *HO-1*, and *NQO1* (*p* < 0.01). These results indicate that PLE can alleviate oxidative stress-induced intestinal and hepatic injury by modulating the mRNA expression levels of key genes in the *PI3K-Akt-Nrf2* signaling pathway, thus preventing oxidative stress-related diarrhea.

#### 3.3.3. Correlation Analysis of Gut Bacteria, Oxidative Stress Biomarkers, Key Targets of the *PI3K-AKT-Nrf2* Signal Pathway, and SCFAs

**Figure 9:**
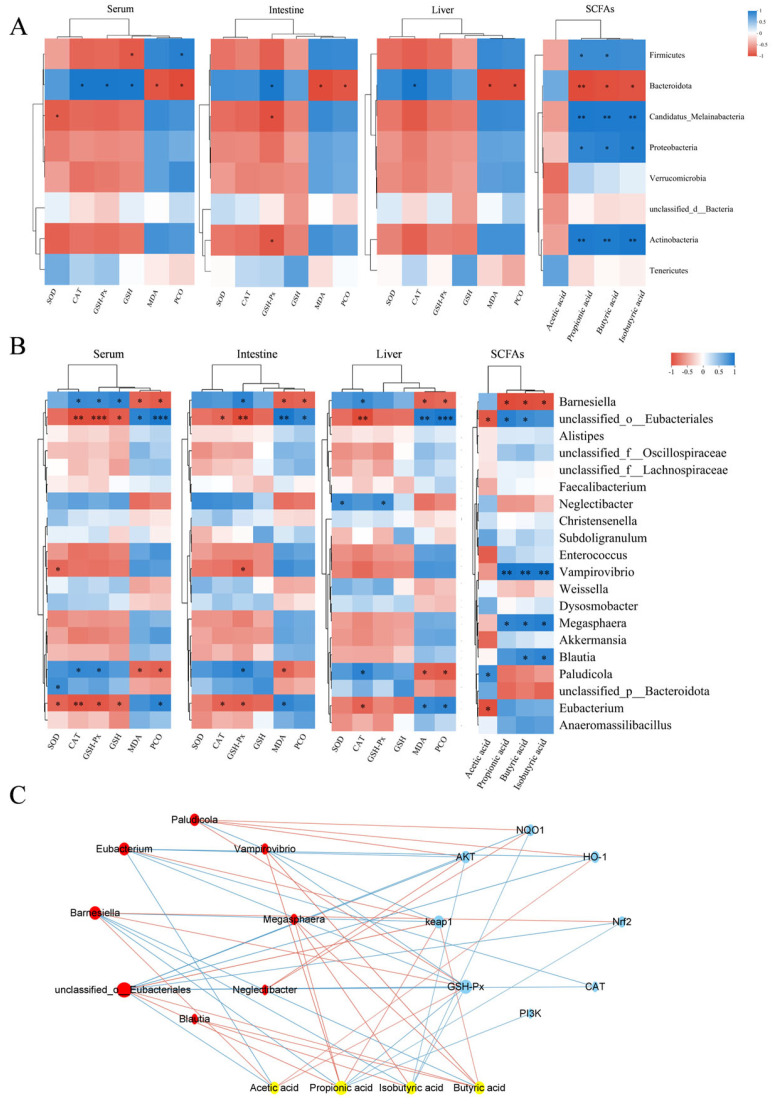
Correlation analysis. (**A**) Heatmap showing the correlation analysis of gut bacteria (phylum level), short-chain fatty acids (SCFAs), and oxidative stress biomarkers. (**B**) Heatmap showing the correlation analysis of gut bacteria (genus level), SCFAs, and oxidative stress biomarkers. In panels A and B, blue represents a positive correlation, while red represents a negative correlation. * indicates *p* < 0.05, ** indicates *p* < 0.01 and *** indicates *p* < 0.001. (**C**) Plot of the combined analysis of bacteria, metabolites, and genes. In panel C, yellow represents SCFAs, blue represents genes, and red represents bacteria. The red lines represent positive correlations, while the blue lines represent negative correlations. The size of the nodes reflects the magnitude of the correlation coefficient.

To investigate the relationship between antioxidant biomarkers (SOD, CAT, GSH-Px, GSH, MDA, PCO) and SCFAs, as well as gut microbiota, Pearson correlation analysis was conducted at both phylum and genus levels. The results are depicted in Figure 9A. At the phylum level, the correlation analysis indicated that Firmicutes were significantly positively correlated with the levels of PCO, propionic acid, and butyric acid, while they were significantly negatively correlated with the levels of GSH in serum. Bacteroidota exhibited a significant positive correlation with CAT and GSH-Px activities, as well as with GSH levels in serum, GSH-Px activities in intestinal tissues, and CAT activities in liver tissues. In contrast, Bacteroidota exhibited significant negative correlations with serum, intestinal, and hepatic MDA and PCO levels, as well as propionic acid, butyric acid, and isobutyric acid concentrations.

At the genus level, the correlation analysis results, depicted in Figure 9B, indicated that *Barnesiella* was significantly positively correlated with CAT and GSH-Px activities and GSH contents in serum, GSH-Px activities in intestinal tissues, and CAT activities in liver tissues. Additionally, *Barnesiella* was significantly negatively correlated with the contents of MDA and PCO in serum, intestinal tissues, and hepatic tissues, as well as with the contents of propionic acid, butyric acid, and isobutyric acid. *Unclassified_o_Eubacteriales* was significantly positively correlated with the contents of MDA and PCO, as well as with the contents of propionic and butyric acids in serum, intestinal tissues, and liver tissues. It was also significantly negatively correlated with GSH contents in serum, CAT and GSH-Px activities in serum and intestinal tissues, and CAT activities and acetic acid contents in liver tissues.

The relationships among gut microbiota, metabolites, and genes were analyzed to investigate their functional impacts. Correlation analysis was performed on the top 20 bacteria, short-chain fatty acids (acetic acid, propionic acid, butyric acid, and isobutyric acid), and key targets of the *PI3K-AKT-Nrf2-Keap1* signaling pathway, as well as their downstream genes. The findings (Figure 9C) indicated that *Barnesiella* and *unclassified_o_Eubacteriales* were significantly correlated with short-chain fatty acids and signaling pathway genes. *Barnesiella* positively correlated with acetic acid, GSH-Px, *Nrf2*, and *HO-1*, and negatively correlated with *Keap1*, propionic acid, butyric acid, and isobutyric acid. In contrast, *unclassified_o_Eubacteriales* exhibited the opposite regulatory pattern, negatively correlating with *AKT*, *NQO1*, and *CAT*. Acetic acid positively correlated with *Barnesiella*, *HO-1*, and *GSH-Px*, while negatively affecting *unclassified_o_Eubacteriales*. Propionic acid positively correlated with *unclassified_o_Eubacteriales* and *Keap1*, but negatively impacted *Barnesiella*, *PI3K*, *Nrf2*, and *GSH-Px*. Butyric acid also positively influenced *unclassified_o_Eubacteriales* and *Keap1*, but negatively impacted *Barnesiella*. Lastly, isobutyric acid negatively correlated with *Barnesiella*, *AKT*, *NQO1*, and *GSH-Px*.

## 4. Discussion

DQ is a widely used bipyridyl non-selective contact herbicide that induces significant cellular OS primarily by affecting redox reactions. Bai et al. [16] demonstrated that broilers treated with 10 mg/kg body weight diquat consistently exhibited OS throughout the experimental period, as evidenced by significantly reduced activities of antioxidant enzymes (SOD and GSH-Px) and clinical manifestations including anorexia and diarrhea. In our study, the DQ group showed a diarrhea rate of 97.7%, confirming the successful establishment of the DQ-induced diarrhea model. Qin et al. [17] reported that dietary supplementation with ellagic acid alleviated diarrhea caused by weaning stress in piglets, which is consistent with the results of the present study. The addition of PLE to the diet significantly reduced DQ-induced OS-related diarrhea, further confirming its preventive effect on poultry diarrhea.

The negative effects of OS on broiler growth performance have been well established, and broiler performance directly impacts farming efficiency. The beneficial effects of plant extracts on growth may be mediated by their abundant bioactive compounds such as polyphenols and flavonoids [18]. In this study, PLE, which is rich in these compounds, likely contributed to the observed improvement in broiler growth performance. Previous studies have shown that resveratrol alleviates LPS-induced declines in production performance [19], while total flavonoids from Artemisia oleifera mitigate lipopolysaccharide-induced reductions in body weight and ADG [20]. In this experiment, supplementation with PLE significantly increased the ADG and reduced the F/G, further supporting its positive effect on broiler growth performance.

The intact intestinal epithelium serves as a crucial barrier between the host and external environment, fulfilling essential protective functions by preventing pathogen invasion and blocking the translocation of harmful molecules and antigens [21]. The intestinal physical barrier consists of intestinal epithelial cells and the intact structures formed by tight junctions [22]. Among these, *Muc2* plays a crucial role in barrier function by forming a protective mucus layer that prevents pathogen invasion and regulates the immune response. Tight junctions, on the other hand, consist of a complex system formed by the interaction of a series of transmembrane proteins (e.g., *Claudins* and *Occludin*) and peripheral proteins (e.g., *ZO-1*) [23]. Our results showed that the mRNA expression levels of *Occludin*, *ZO-1*, *Claudin-1*, and *MUC2* were significantly higher in the PLE group compared to those in the DQ group. This finding indicates that PLE significantly improved intestinal barrier function and enhanced overall intestinal health. The study by Ingegneri et al. [24] also demonstrated that citrus pomace, rich in flavonoids and phenolic acids, could prevent oxidative stress-induced intestinal barrier damage. The villus height-to-crypt depth ratio serves as a critical morphological indicator for evaluating intestinal health and functional capacity. Increased values of this ratio generally correlate with enhanced nutrient absorption efficiency and improved epithelial renewal processes, reflecting better intestinal homeostasis. [25]. Our results showed that the PLE group significantly increased the ratio of villus height to crypt depth, further demonstrating that PLE supports the maintenance of intestinal structural integrity and optimizes its function.

ALT and AST are two key liver enzymes. These enzymes are primarily located in hepatocytes, and they are released into the bloodstream when hepatocytes are damaged or diseased. Elevated serum levels of ALT and AST can reflect the extent of hepatocyte damage [26]. In our experiment, the addition of PLE to the diet reduced serum ALT and AST activities, suggesting that PLE can prevent DQ-induced liver injury. The study by Huang et al. [27] indicated that chlorogenic acid and flavonoids, contained in the leaf extract of C. juncea, were able to reduce plasma levels of AST and ALT, which is consistent with our findings.

T-AOC represents the integrative capacity of the body’s non-enzymatic antioxidant defense system. A significant portion of the total antioxidant capacity of cells is attributed to the endogenous antioxidant system, which includes endogenously synthesized antioxidant enzymes (e.g., SOD, GSH-Px, and CAT) as well as antioxidants such as vitamin E and GSH. These naturally occurring antioxidant enzymes degrade highly unstable ROS through their respective enzymatic reaction systems, thereby maintaining the oxidative and antioxidant balance in vivo [28]. SOD, an enzyme widely distributed in all aerobic organisms, catalyzes the conversion of O_2_^•−^ to O_2_ and H_2_O_2_, serving as the first line of defense against OS [29]. CAT breaks down H_2_O_2_ into O_2_ and H_2_O, preventing oxidative damage. GSH, a crucial endogenous intracellular antioxidant, acts as a substrate for GSH-Px, which reduces H_2_O_2_ or organic peroxides to water [30]. MDA, a key by-product of lipid peroxidation, is widely used as a biomarker for OS and cell membrane damage, with its level reflecting the extent of oxidative damage to cells [31]. Our results showed that T-AOC levels, antioxidant enzyme activities, and GSH contents were significantly increased, while MDA contents were significantly decreased in broilers following the addition of PLE. These findings are consistent with those of Liu [32], who studied the antioxidant properties of paeonia petals and demonstrated that flavonoid extracts from paeonia petals increased the activities of SOD, GSH-Px, and CAT in serum and liver, while reducing MDA contents. Similarly, another study on the antioxidant effects of flavonoids reported that flavonoid extracts from grasshoppers enhanced antioxidant enzyme activities and reduced MDA content in rats [33]. Daud et al. [34] similarly demonstrated that Acacia polyphenol extract increased GSH contents in the liver, which is in line with our findings. Protein oxidation can be directly induced by ROS or indirectly through oxidative byproducts such as MDA. In OS studies, the detection of PCO content not only reflects the extent of oxidative protein damage but also serves as an indicator of impaired cellular function [35]. In our study, the PLE group significantly mitigated the DQ-induced elevation in PCO levels, suggesting that PLE has a substantial preventive and protective effect against OS-induced protein damage.

The gut microbiota is the largest symbiotic ecosystem within the host, influencing a wide range of physiological functions by regulating immune and metabolic pathways, and playing a crucial role in maintaining homeostasis. OS diarrhea is closely associated with an imbalance in gut flora. In this study, we performed full-length 16S rRNA sequencing of cecal contents, which have a high microbial density, to investigate the effects of DQ-induced acute OS on the composition of the intestinal flora and its role in OS diarrhea, as well as the impact of PLE supplementation in restoring gut flora balance. Our analysis of phylum-level species composition revealed that broiler gut bacteria were predominantly dominated by Firmicutes and Bacteroidetes, which is consistent with the findings of Broom et al. [36], who analyzed the cecal microbial composition in broilers. Firmicutes serve as a source of energy and are involved in the host’s material and energy metabolic cycles. Meanwhile, Bacteroidetes participate in the metabolism of carbohydrates, polysaccharides, and other nutrients, promoting nutrient absorption. The abundance of Firmicutes and Bacteroidetes communities in the CON group was 48.45% and 45.55%, respectively. After DQ stimulation, the abundance of Firmicutes up-regulate to 71.66%, while Bacteroidetes down-regulate to 14.34%. This change in flora composition was associated with an elevated F/B ratio, indicating that an imbalance in the intestinal flora may be a potential cause of OS diarrhea. After PLE supplementation, this trend was reversed, and the flora composition was restored to levels similar to those of the CON group. Compared to the DQ group, PLE significantly reduced the F/B ratio, suggesting its potential to prevent DQ-induced intestinal flora imbalance and alleviate OS diarrhea. These findings are consistent with previous studies, such as Wu [37], where the addition of curcumin to broiler diets prevented DQ-induced gut microbiota imbalance by regulating the abundance of Bacteroidetes and Firmicutes phyla.

Analysis at the genus level can reveal specific microbial genus variations, helping us identify key pathogens and beneficial bacteria, thereby providing a more precise understanding of the dynamic changes in the gut microbiota under OS conditions and its functional roles. *Barnesiella*, a member of Bacteroidetes, has been reported to promote the conversion of polyphenols and flavonoid compounds into SCFAs, such as acetate and butyrate, through deglycosylation in the gut microbiota. As a target bacterium for dietary polyphenols, *Barnesiella* not only eliminates harmful bacteria in the intestine but also produces SCFAs that are beneficial to gut health [38]. In our study, the abundance of *Barnesiella* was significantly down-regulated in the DQ group and significantly up-regulated in the PLE group. Zhao et al. [39] demonstrated that florfenicol-induced reduction in *Barnesiella* abundance in broilers under OS was alleviated by dietary supplementation with Lactobacillus plantarum P8, which significantly up-regulated *Barnesiella* abundance, similar to our results. Another study also showed that dietary inclusion of konjac flour alleviated OS and up-regulated *Barnesiella* abundance in sows during gestation [40]. Additionally, our results showed down-regulation in the abundance of *unclassified_f__Oscillospiraceae* in the PLE group. It has been reported that an up-regulation in the abundance of this bacterium is significantly correlated with butyric acid production. A similar study found that green brick tea extract ameliorated gut microbial-mediated metabolic disorders in high-fat mice and reduced the abundance of *unclassified_f__Oscillospiraceae*. Therefore, a moderate reduction in the abundance of *unclassified_f__Oscillospiraceae* in the PLE group may help maintain overall microbiota balance and prevent the overgrowth of specific bacterial groups, thereby promoting gut health. *Unclassified_o__Eubacteriales* are typically considered conditionally pathogenic bacteria. *Eubacteriales* are significantly up-regulated in abundance in patients with inflammatory bowel disease (IBD) and may regulate intestinal inflammation and immune responses by metabolizing bile acids and SCFAs [41]. In our study, OS occurred in the organisms of the DQ group, and the up-regulated abundance of *Eubacteriales* may represent a protective response. In contrast, in the PLE group, the abundance of *Eubacteriales* was down-regulated due to the lower levels of OS. These results further support the positive role of PLE in regulating the balance of intestinal flora and alleviating OS diarrhea.

SCFAs are the main products of soluble fiber fermentation by gut microbiota and are utilized by intestinal microorganisms. They play an important role in maintaining health and in the development of diseases [42]. SCFAs mitigate OS, inflammatory responses, and metabolic disorders [43]. Acetic acid has been reported to improve the viability of pancreatic islets and the mouse insulinoma cell line MIN6 under OS conditions by enhancing ROS metabolism [44]. In our study, acetic acid content was significantly lower in the DQ group compared to the CON group, suggesting that OS inhibits acetic acid production, possibly due to an imbalance in the gut microbiota, particularly a decrease in the abundance of acetic acid-producing bacteria. According to the genus level analysis, the decrease in Barnesiella abundance in the DQ group may be one of the reasons for the decrease in acetic acid. In contrast, in the PLE group, the acetic acid content was significantly elevated, indicating that PLE was able to restore the ability of acetic acid production by regulating the intestinal flora. The significant increase in propionic acid, butyric acid, and isobutyric acid contents in the DQ group may be due to OS-induced intestinal inflammation and microbial imbalance, which resulted in the overgrowth of certain short-chain fatty acid-producing bacteria, consistent with the genus-level findings. In the DQ group, Eubacterium spp., capable of producing SCFAs, and *Alistipes* showed increased abundance. The levels of all three acids were significantly reduced under PLE intervention, approaching or reverting to the levels seen in the CON group. Our results are in line with those of Liu [45], who reported that polyphenol extracts from Genbaku maple leaves reduced LPS-induced elevated levels of propionic and butyric acids in the cecal contents of broilers. Taken together, PLE demonstrated significant potential in alleviating OS and regulating the metabolism of SCFAs, providing a foundation for further research into the mechanisms underlying the action of plant extracts in promoting intestinal health.

In our study, PLE targeted *AKT1*, which plays a crucial role in cell survival and anti-OS via the *PI3K-AKT* signaling pathway. This pathway promotes cell survival, proliferation, and the expression of antioxidant genes. These combined actions help cells respond to and adapt to OS, maintaining normal cellular and systemic functions. Based on network pharmacology pathway prediction, we investigated the role of the *PI3K-AKT* signaling pathway in activating the Nrf2-Keap1 pathway to regulate OS. Activation of the *PI3K-AKT* signaling pathway promotes the release of *Nrf2* from the *Keap1* complex into the nucleus, initiating the transcription of antioxidant genes. Upon *Nrf2* activation, downstream genes such as *HO-1* and *NQO1* are transcribed and translated. These antioxidant enzymes play a critical role in scavenging ROS and protecting cells from oxidative damage [46]. In the DQ group, we observed a significant decrease in the expression of the *PI3K-AKT* signaling pathway in both the intestine and liver. This result suggests that OS has an inhibitory effect on the *PI3K-AKT* signaling pathway in these organs. The PI3K-AKTT signaling pathway plays a crucial role in regulating cell survival, proliferation, and metabolism, and its downregulation may contribute to cellular resistance to OS [47]. Meanwhile, the expression of *Nrf2* in both the intestine and liver was significantly reduced, while the expression of Keap1 was significantly increased. This change further indicates the inhibitory effect of OS on the *Nrf2-Keap1* signaling pathway in these organs. Nrf2 is a critical transcription factor that regulates the cellular antioxidant response, whereas elevated expression of Keap1, a negative regulator of Nrf2, inhibits the nuclear translocation of *Nrf2* and reduces the expression of antioxidant genes. Our results also showed that the expression of intestinal and hepatic antioxidant enzymes, such as *HO-1* and *NQO1*, which are downstream of the *Nrf2-Keap1* pathway, was significantly decreased, suggesting that OS severely impairs the cellular antioxidant defense mechanism. The expression of the *PI3K-AKT* signaling pathway was significantly elevated in the PLE group compared to the DQ group. This suggests that PLE was able to restore the activity of the *PI3K-AKT* signaling pathway, thereby enhancing cell survival and stress resistance. Additionally, the expression of *Nrf2* was significantly elevated, while the expression of Keap1 was significantly reduced in the PLE group, indicating that PLE could activate the expression of antioxidant genes by regulating the *Nrf2-Keap1* signaling pathway and promoting the nuclear translocation of *Nrf2*. Consequently, the expression of downstream antioxidant enzymes, such as *HO-1* and *NQO1*, was significantly increased, consistent with previous research showing that hesperidin upregulates antioxidant enzyme levels through the *PI3K-AKT-Nrf2* pathway, alleviating oxidative damage induced by excessive ROS production [48].

Correlation analysis of OS markers and metabolites with gut microbiota revealed that at the phylum level, an increase in the abundance of Firmicutes was associated with decreased antioxidant capacity, increased oxidative damage, and reduced acetic acid content, whereas an increase in Bacteroidetes contributed to enhanced antioxidant defenses, reduced oxidative damage, and increased acetic acid content. Among the genera showing significant changes, *Barnesiella* (belonging to the phylum Bacteroidetes) and *unclassified_o_Eubacteriales* (belonging to the phylum Firmicutes) were identified. *Unclassified_o_Eubacteriales* showed a negative correlation with antioxidant enzymes and acetic acid, and a positive correlation with lipid and protein oxidation levels, while *Barnesiella* exhibited the opposite trend. Thus, PLE may prevent DQ-induced OS and intestinal dysbiosis by decreasing the abundance of Firmicutes and increasing the abundance of Bacteroidetes. This modulation is likely mediated through regulation of these specific genera.

The association analysis among bacteria, metabolites, and genes further elucidated the mechanism through which PLE exerts its antioxidant effects. It revealed a close relationship between the gut microbiota *Barnesiella* and *unclassified_o_Eubacteriales*, SCFAs, and pathway-related genes. Therefore, PLE alleviates OS-induced diarrhea in broilers by regulating the *PI3K-AKT-Nrf2-Keap1* signaling pathway, modulating the antioxidant effects of gut microbiota (*Barnesiella* and *unclassified_o_Eubacteriales*), and influencing the production of SCFAs (acetic acid, propionic acid, butyric acid, and isobutyric acid).

## 5. Conclusions

PLE can improve the growth performance and feed efficiency of broilers, as well as enhance the indices of liver, kidney, and bursa. PLE prevents OS-induced diarrhea in broilers by reducing the levels of OS biomarkers, such as MDA and PCO, while increasing the activity of antioxidant enzymes, including SOD, CAT, and GSH-Px. Furthermore, PLE upregulates the mRNA expression levels of intestinal proteins, regulates the balance of gut microbiota, and increases the contents of SCFAs. Through a comprehensive analysis, we demonstrated that PLE facilitates gene-bacteria-metabolite interactions through the “bacteria–intestine–liver” axis, regulates the *PI3K-AKT-Nrf2 signaling* pathway, and subsequently modulates OS biomarkers (SOD, CAT, GSH-Px, MDA, and PCO) to exert its antioxidant effects, thereby effectively preventing diarrhea induced by oxidative stress. This study is the first to show that *Barnesiella* and *unclassified_o_Eubacteriales* are closely associated with OS, providing a novel theoretical framework for understanding the mechanism of antioxidant action.

## Figures and Tables

**Table 1:** Sequences of RT-qPCR primers.

Gene	Primer Sequence (5′-3′)
*MUC2*	F: AATGCTGAGTTCTTGCCTAA
	R: TGTTGCAGTTCATATCCTGGT
*ZO-1*	F: CCACTGCCTACACCACCATCTC
	R: CGTGTCACTGGGGTCCTTCAT
*Occludin*	F: CCTCATCGTCATCCTGCTCT
	R: GGTCCCAGTAGATGTTGGCT
*Claudin 1*	F: GGGGACAACATCGTGACCG
	R: AGGAGTCGAAGACTTTGCACT
*SOD*	F: TTGTCTGATGGAGATCATGGCTTC
	R: TGCTTGCCTTCAGGATTAAAGTGA
*CAT*	F: GTTGGCGGTAGGAGTCTGGTCT
	R: GTGGTCAAGGCATCTGGCTTCTG
*GSH-Px*	F: AACCAATTCGGGCACCAG
	R: CCGTTCACCTCGCACTTCTC
*PI3K*	F: CGGATGTTGCCTTACGGTTGT
	R: GTTCTTGTCCTTGAGCCACTGAT
*AKT*	F: TCACTCCTCCTGACCAAGATGACAG
	R: GCGGTTCCACTGGCTGAATAGG
*Nrf2*	F: GATGTCACCCTGCCCTTAG
	R: CTGCCACCATGTTATTCC
*Keap1*	F: CTGCTGGAGTTCGCCTACAC
	R: CACGCTGTCGATCTGGTACA
*HO-1*	F: CACTCTGGAGATGACACCTGAG
	R: GTGTTCCTCTGTCAGCATCACC
*NQO1*	F: TCGCCGAGCAGAAGAAGATTGAAG
	R: CGGTGGTGAGTGACAGCATGG
*GADPH*	F: GACCACTGTCCATGCCATCA
	R: AACTGAGCGGTGGTGAAGAG

**Table 2:** Effects of PLE on growth performance and relative organ weights of broilers.

Item	CON	DQ	PLE	*p*
ADG(g/d)	84.59 ± 1.14 ^bc^	84.21 ± 1.75 ^c^	90.62 ± 1.60 ^a^	<0.01
ADFI(g/d)	114.11 ± 3.59	112.64 ± 3.17	108.23 ± 3.54	>0.05
F/G(g/g)	1.36 ± 0.03 ^a^	1.35 ± 0.03 ^a^	1.19 ± 0.02 ^b^	<0.01
Liver index (mg/g)	20.12 ± 0.41 ^b^	20.20 ± 0.56 ^b^	23.92 ± 0.64 ^a^	<0.001
Kidney index (mg/g)	1.22 ± 0.06 ^b^	1.18 ± 0.06 ^b^	2.09 ± 0.07 ^a^	<0.001
Spleen index (mg/g)	1.04 ± 0.12	1.08 ± 0.08	1.07 ± 0.27	>0.05
Thymus index (mg/g)	4.45 ± 0.12 ^a^	3.07 ± 0.07 ^b^	4.91 ± 0.41 ^a^	<0.001
Thymus index (mg/g)	0.58 ± 0.08	0.54 ± 0.02	0.54 ± 0.01	>0.05

Note: In the same column, identical superscript letters denote no significant difference (*p* > 0.05), whereas different superscript letters signify a significant difference (*p* < 0.05), as illustrated in the table below.

**Table 3:** Chemical composition of PLE.

Category	Compound Name	Molecular Formula
Polyphenolic compounds	Chlorogenic acid	C_16_H_18_O_9_
	Pyrogallol	C_6_H_6_O_3_
	Syringic acid	C_9_H_10_O_5_
	Caulophyllogenin	C_6_H_6_O_2_
	Hydroxycinnamic acid	C_9_H_8_O_3_
	(−)-catechin	C_15_H_14_O_6_
	Ellagic acid	C_14_H_6_O_8_
	Resveratrol	C_14_H_12_O_3_
	Piceatannol	C_14_H_12_O_4_
	Ferulic acid	C_10_H_10_O_4_
	5-Hydroxyferulate	C_10_H_10_O_5_
	2-Hydroxycinnamic acid	C_9_H_8_O_3_
Flavonoids	(+/−)-Naringenin	C_15_H_12_O_5_
	Kaempferol	C_15_H_10_O_6_
	2-(3-Hydroxyphenyl)-6-methyl-4H-chromen-4-one	C_6_H_12_O_3_
	Quercetin	C_15_H_10_O_7_
	Taxifolin	C_15_H_12_O_7_
	Xanthurenic acid	C_10_H_7_NO_4_
	Genistein	C_15_H_10_O_5_
Organic acids	2-isopropylmalic acid	C_7_H_12_O_5_
	Hexahydro-1,3,4,5-tetrahydroxybenzoic acid	C_6_H_8_O_2_
	Cis-4-coumaric acid	C_9_H_8_O_3_
	Benzoic acid	C_7_H_6_O_2_
	Lauric acid	C_12_H_24_O_2_
	(S)-(−)-PERILLIC ACID	C_10_H_14_O_2_
	Azelaic acid	C_9_H_16_O_4_
Organic acids	Oxypaeoniflorin	C_23_H_28_O_12_
	6′-O-Glucopyranosylalbiflorin	C_29_H_38_O_16_
	Paeoniflorin	C_23_H_28_O_11_
	Galloylalbiflorin	C_30_H_32_O_15_
	Benzoylpaeoniflorin	C_30_H_32_O_12_
	Astragalin	C_21_H_20_O_11_
	Salidroside	C_14_H_20_O_7_
	Cyanidin3-O-rutinoside	C_27_H_30_O_15_
	albiflorin	C_23_H_28_O_11_
	Picroside II	C_23_H_28_O_13_
Other compounds	(S)-10-Hydroxycamptothecin	C_20_H_16_N_2_O_5_
	Coumarin	C_9_H_6_O_2_
	4-Isopropylbenzaldehyde	C_10_H_12_O
	Caprolactam	C_6_H_11_NO
	L(−)-Carvone	C_10_H_14_O
	Pleuromutilin	C_22_H_34_O_5_
	CARMINIC ACID	C_22_H_20_O_13_
	Coniferyl alcohol	C_10_H_12_O_3_
	Costunolide	C_15_H_20_O_2_
	Genistein	C_15_H_10_O_5_

## Data Availability

The data that support the findings of this study are available on request from the corresponding author.
